# Supplementary material for: ER calcium depletion as a key driver for impaired ER-to-mitochondria calcium transfer and mitochondrial dysfunction in Wolfram syndrome
Source: Nat Commun. 2024 Jul 21;15:6143. doi: 10.1038/s41467-024-50502-x (PMC11271478; doi:10.1038/s41467-024-50502-x)
Supplement: Supplementary file 1 — Supplementary Information [file 41467_2024_50502_MOESM1_ESM.pdf]

SUPPLEMENTARY INFORMATION

ER calcium depletion as a key driver for impaired ER-to-mitochondria calcium transfer and mitochondrial dysfunction in Wolfram syndrome

Mailis Liiv, Annika Vaarmann, Dzamilja Safiulina, Vinay Choubey, Ruby Gupta, Malle Kuum, Lucia Janickova, Zuzana Hodurova, Michal Cagalinec, Akbar Zeb, Miriam A. Hickey, Yi-Long Huang, Nana Gogichaisvili, Merle Mandel, Mario Plaas, Eero Vasar, Jens Loncke, Tim Vervliet, Ting-Fen Tsai, Geert Bultynck, Vladimir Veksler, Allen Kaasik

Supplementary Methods

Supplementary Figures

- Supplementary Fig. 1. shRNAs against *Cisd2*, *IP_3R1, IP_3R3*, and *RYR2* efficiently suppress the expression of respective mRNAs.
- Supplementary Fig .2. Effect of SERCA activators and inhibitor on ER Ca^{2+} uptake and Ryanodine receptor inhibitors on ER Ca^{2+} release.
- Supplementary Fig. 3. Single-cell expression analysis of SERCA2, RYR2, and IP3R1 in Wfs1 or Cisd2 knockdown neurons.
- Supplementary Fig. 4. Decreased resting mitochondrial Ca^{2+} levels in the axons of WFS1 and CISD2 deficient neurons as measured by higher affinity mitochondria-targeted Ca^{2+} indicator GCepia2.
- Supplementary Fig. 5. Wt, K836N and 864K, but not A684V and P724L WFS1, were able to restore resting mitochondrial Ca^{2+} levels in the axons of WFS1-deficient neurons.
- Supplementary Fig. 6. Overexpression of IP_3R1 but not its pore-dead mutant restores cytosolic Ca^{2+} levels in the axons of WFS1 deficient neurons.
- Supplementary Fig. 7. Proximity Ligation Assay (PLA) reveals close proximity of WFS1 and CISD2 in neurons.
- Supplementary Fig. 8. Computational docking model for human CISD2 and WFS1.
- Supplementary Fig. 9. Mitochondrial length is restored in WFS1-deficient neurons when overexpressing CISD2 (A) and in CISD2-deficient neurons when overexpressing WFS1 (B).
- Supplementary Fig. 10. Suppressed axonal growth in WFS1- or CISD2-deficient neurons is restored when expressing SERCA2b.
- Supplementary Fig. 11. Effects of different compounds on mitophagy, mitochondrial density, ATP/ADP ratio, mitochondrial length, and axonal development.

Supplementary References

Supplementary Methods

Supplementary Table 1. List of Reagents and Resources

Reagent or Resource	Source	Identifier (Cat#)
Plasmids		
Fluorescent sensors		
mito G-CEPIA2	Addgene	58218
mito G-CEPIA3	Addgene	58219
ER-GCamp6-210	Addgene	86919
PercevalHR	Addgene	49082
EGFP	Clontech	6085–1
SoNar	Gift from Dr. Yi Yang	
Syn-jGCaMP7	Addgene	104489
Camk2-Ace-8aa-mScarlet	Addgene	129702
mtKeima	Amalgaam	AM-V0251
Peredox-mCitrine	Addgene	32386
Syn-DsRedExpress	Addgene	22907
DsRed2-Mito	Clontech	632421
mCherry-LifeAct7	Addgene	54491
iRFP720	Addgene	45461
MAMtracker-Luc	Gift from Dr. Koji Yamanaka	
Overexpression plasmids		
CISD2-YPet	Generated in our lab	
CISD2-flag	Generated in our lab	
CISD2-myc-flag	Origene	RC267131
IP_3R1 Wt and D2550A	Gift from Dr. David Yule	
IP_3R1 active fragment	DF/HCC DNA Resource Core	MmCD00312368
SERCA2b	Addgene	75188
PDK1	Addgene	111672
Miro1	Addgene	47888
Phospholamban	Origene	RC202712
PDK1	Addgene	20564
PDP2	Sino Biological	158-HG24215-CF
WFS1 mRFP-N2 (891)	Addgene	62018
WFS1 pEGFP	Addgene	62051
WFS1-myc	Generated in our lab	
WFS1-flag	Addgene	13011
WFS1 P724L-flag	Addgene	13012
WFS1 other mutants	Generated in our lab	
GRP75	Gift from Gyorgy Szabadkai	
shRNA-s		
Cisd2 shRNA	SABiosciences	KR47927N

Cisd2 siRNA	Sigma-Aldrich	SASI_Rn02_00358487 XM_001077768
IP_3R1 shRNA	SABiosciences	KR42677N
IP_3R3 shRNA	SABiosciences	KR45319N
RYR2 shRNA	Origene Technologies, USA	TR713964
Wfs1 shRNA	SABiosciences	KR46208N
Wfs1 siRNA	Sigma-Aldrich	SASI_Rn02_00265296 NM_031823
Primers		
CISD2_F	CTTGCAGTTCGTCCATTCTT	Generated in our lab
CISD2_R	ATCGCCTGTCAACTCATTGT	Generated in our lab
IP3R1_F	ATG AGC ACA GGA AGA AGC AG	Generated in our lab
IP3R1_R	ACC TCT GCT GCC AAG TAA TG	Generated in our lab
IP3R3_F	CAA GTA CCT CAC CGT GAA CA	Generated in our lab
IP3R3_R	CGA CTT TAT CTC CCA CGA CG	Generated in our lab
RyR2_F	CAC GCC ATA CTA CTA CGC CAT	Generated in our lab
RyR2_R	CTG TTG CCG TAT GAC AAG TGC	Generated in our lab
HPRT_F	GCG AAA GTG GAA AAG CCA AGT	Generated in our lab
HPRT_R	GCC ACA TCA ACA GGA CTC TTG TAG	Generated in our lab
CycA_F	GAG CAC TGG GGA GAA AGG AT	Generated in our lab
CycA_R	CTT GCC ATC CAG CCA CTC AG	Generated in our lab
Cell Culture		
B-27™ Plus supplement	ThermoFisher Scientific	A3582801
B-27™ supplement	ThermoFisher Scientific	17504044
Basal Medium Eagle (BME)	ThermoFisher Scientific	41010109
DMEM, high glucose, GlutaMAX™ Supplement, pyruvate	ThermoFisher Scientific	31966021
Lipofectamine® 2000	ThermoFisher Scientific	11668019
Neurobasal™-A with or without fenol red	ThermoFisher Scientific	10888022 or 12349015 (without fenol red)
N-TER Nanoparticle siRNA Transfection System	Sigma-Aldrich	N2913
Opti-MEM I Reduced Serum Medium	ThermoFisher Scientific	11058021
Poly-L-lysine hydrobromide	Sigma-Aldrich	P6282
Chemicals		
(RS)-3,5-DHPG	Tocris	342
Azymolene sodium salt	Cayman Chemical	16462
CDN1163	Tocris	5869
CGP37157	Tocris	1114
Liraglutide	Tocris	6517
Pyruvate dehydrogenase inhibitor (CPI613)	Tocris	5348
RYR-Calstabin Interaction Stabilizer (RyCal S107)	Millipore, Sigma-Aldrich	500469
Thapsigargin	Invitrogen	T7459
BHQ	Tocris	1236
FCCP	Tocris	453
JC-10 [Enhanced JC-1], Ultra Pure	Enzo	ENZ-52305
Mag-Fluo-4, AM	Invitrogen	M14206

Antibodies		
Rabbit polyclonal anti-WFS1 (1:1000)	Proteintech	26995-1-AP
Rabbit polyclonal anti-WFS1 (1:1000)	Thermo Fisher Scientific	PA5-76065
Mouse monoclonal anti-CISD2, clone 3D7A3 (1:1000)	Proteintech	66082-1-Ig
Rabbit polyclonal anti-CISD2 (1:1000)	Proteintech	13318-1-AP
Rabbit polyclonal anti-CISD2 (1:1000)	Abclonal	A5231
Rabbit polyclonal anti-SERCA2 (1:1000 WB, 1:200 IC)	Proteintech	27311-1-AP
Mouse monoclonal anti-SERCA2, clone IID8 (1:1000)	Invitrogen	MA3-910
Mouse monoclonal anti-RYR, clone 34C (1:20 IC)	DSHB	34C
Mouse monoclonal anti-RyR2, clone C3-33 (1:1000)	Thermo Fisher Scientific	MA3-916
Rabbit polyclonal anti-RyR2 (1:1000)	Generated in our lab	
Rabbit polyclonal anti-IP3R (1:200 IC)	Proteintech	19962-1-AP
Mouse monoclonal anti-β-actin, clone AC-74 (1:2000)	Sigma-Aldrich	A228
Rabbit anti-MYC (1:3000)	Abcam	ab9106
Rabbit anti-FLAG (1:1000)	Sigma-Aldrich	F7425
Mouse anti-MYC (1:500)	Thermo Fisher Scientific	R950-25
Mouse anti-GFP (1:1000)	Roche	11814460001
Goat anti-Rabbit Alexa Fluor™ Plus 488 (1:1000 IC)	Invitrogen	A32731
Goat anti-Mouse Alexa Fluor™ 488 (1:1000 IC)	Invitrogen	A11001
Goat anti-mouse IRDye 680LT (1:2000)	LI-COR Biosciences	926-68020
Goat anti-mouse IRDye 800CW (1:5000)	LI-COR Biosciences	925-32210
Goat anti-rabbit IRDye 800CW (1:5000)	LI-COR Biosciences	926-32211
Goat anti-rabbit IRDye 680LT (1:2000)	LI-COR Biosciences	926-68021
Donkey anti-mouse MINUS 100 (according to manufacturer)	Olink Bioscience	92004-0100
Donkey anti-rabbit PLUS 100 (according to manufacturer)	Olink Bioscience	92002-0100
Other		
Duolink In Situ (Proximity Ligation Assay)	Olink Bioscience	92002-0100
		92004-0100
		92008-0100
GFP-Trap Agarose	Chromotek	gta-20
Odyssey blocking buffer	LI-COR Bioscience	927–40000
DC™ Protein Assay Reagent A, B, S	Bio-Rad	500-0113; 500-0114; 500-0115
Nano-Glo® Live Cell Assay System	PROMEGA	N2012

Co-immunoprecipitation and Western Blotting

For immunoprecipitation of overexpressed WFS1, transfected HEK293 cells overexpressing EGFP-WFS1 and CISD2-myc were collected in ice-cold PBS and pelleted at 2000g for 5 min at 4°C. Then, cells were lysed in ice-cold IP lysis buffer containing 25 mM Tris-HCl pH 7.2, 150 mM NaCl, 5 mM $MgCl_2$, 1% NP-40, 5% glycerol and protease inhibitor cocktail (Roche). Cell lysates were passed through a 26G needle altogether 10 times each during incubation on ice for 30 min with shaking at 350 rpm. The insoluble fraction was removed by repeated centrifugation first at 30 000g for 15 min at 4°C and then 55 000g for 15 min at 4°C. The protein concentrations of the lysates were estimated using DC protein

assay method (Bio-Rad) according to the manufacturer's protocol. EGFP-WFS1 was then immunoprecipitated using the GFP-Trap (ChromoTek). Diluted lysate-supernatant was added to 25 µL of equilibrated GFP-Trap Agarose beads and incubated on a rotary shaker for 2 h at 4°C. The beads were washed twice with ice-cold wash buffer and then twice with wash buffer with increased salt concentration. Beads were resuspended in 2x Lane marker reducing sample buffer (ThermoFisher Scientific) in PBS, boiled at 95°C for 10 min and the supernatant was collected by centrifugation at 2500g for 2 min at 4°C.

Equivalent amounts of proteins were then resolved on 4-20% gradient (Bio-Rad) polyacrylamide gel by SDS-PAGE. A cold-block electrophoretic transfer system with 0.1 M Tris-base, 0.192 M glycine, and 10% (w/w) methanol was used to transfer resolved proteins to Immobilon® PSQ membranes (ISEQ00010, Millipore). The membranes were blocked using Odyssey blocking buffer (LI-COR Bioscience) at room temperature for 1 h. After blocking, the membrane with electrophoretically separated proteins was incubated overnight, first with rabbit anti-myc 1:3000 (Abcam), next with mouse anti-GFP 1:1000 (Roche), and finally with mouse anti-β-actin 1:2000 (Sigma-Aldrich). Incubations were followed by 3x washing in Tris Buffered Saline containing 0.1% Tween-20 followed by incubation with the appropriate secondary antibody, either goat anti-rabbit IRDye 800CW 1:5000, goat anti-mouse IRDye 680LT 1:20000 or goat anti-mouse IRDye 800 CW 1:5000 (all from LI-COR Biosciences) for 30 min at room temperature. The immunoreactive bands were detected with Odyssey Infrared Imaging System (LI-COR Bioscience).

For immunoprecipitation of endogenous CISD2 and SERCA2, cell lysates from non-transfected HEK cells were prepared as described above, except for the IP lysis buffer composition (20 mM HEPES pH 7.4, 150 mM NaCl, 5 mM EDTA, 1% Triton-X-100, 10% glycerol, protease inhibitor cocktail and 1 mM Phenylmethanesupfonyl fluoride (PMSF, P7626 Sigma-Aldrich). 500 µl of lysate (1-3 mg of protein) was incubated overnight at 4°C with head over head mixing with mouse monoclonal anti-CISD2 (Proteintech 66082-1-Ig, 8 µg) or mouse monoclonal anti-SERCA2 (Invitrogen, IID8, MA3-910, 5 µg) antibody or mouse IgG1 (Abcam, ab18443, 5-8 µg). Next day, samples were transferred into the Pierce™ spin columns (Thermo Scientific, 69725) and 80 µL of equilibrated rec-Protein G-Sepharose® 4B (Invitrogen, 101242) beads (50% suspension in IP lysis buffer) were added. Samples were further incubated for 3 h with head over head mixing at 4°C. Beads were then washed 5 times with IP lysis buffer, and immunoprecipitates were eluted twice in 40 µl of 200 mM glycine pH 2.5 at room temperature with shaking at 500 rpm for 1 min, centrifuged for 2 min at 2500g and pH of eluate was neutralized with 4 µl of 1M Tris pH 10.4. Eluate was immediately mixed with LI-COR Protein Sample Loading Buffer (4×, LI-COR Bioscience 928-40004) supplemented with 2-Mercaptoethanol (M6250 Sigma-Aldrich), according to the LI-COR's protocol and samples were incubated at room temperature for 10 minutes before loading on polyacrylamide gel.

Equivalent amounts of proteins were then resolved on 4-20% gradient (Bio-Rad 4561094) polyacrylamide gel by SDS-PAGE. A cold-block electrophoretic transfer system with 0.1 M Tris-base, 0.192 M glycine, and 10-20 % (w/w) methanol was used to transfer resolved proteins to Immobilon® FL membranes (IPFL20200, Millipore). The membranes were blocked using Odyssey Intercept® (TBS) Blocking Buffer (LI-COR Bioscience, 927-60001) at room temperature for 1 h. After blocking, the

membrane with electrophoretically separated proteins was incubated overnight with rabbit anti-WFS1 1:1000 (Proteintech), rabbit anti-CISD2 1:1000 (Proteintech), mouse anti-SERCA2 1:1000 (Invitrogen) or rabbit anti-SERCA2A 1:1000 (Proteintech). Incubations were followed by 3x washing in Tris Buffered Saline containing 0.1% Tween-20 followed by incubation with the appropriate secondary antibody, either goat anti-rabbit IRDye 800CW 1:5000, goat anti-mouse IRDye 680LT 1:5000 or goat anti-mouse IRDye 800 CW 1:5000 (all from LI-COR Bioscience) for 45 min at room temperature. The immunoreactive bands were detected with Odyssey Infrared Imaging System.

For co-immunoprecipitation of RyR interacting proteins, whole brain lysates from six-month old mice were obtained by incubating the brains with lysis buffer (pH 7.5, 50 mM Tris-HCl, 100 mM NaCl, 2 mM EDTA, 50 mM NaF, 1 mM Na_3VO_4, 1% CHAPS and protease inhibitor coctail (Roche, Basel, Switzerland)) followed by mechanical homogenizing using an Eppendorf douncer. Once fully homogenized the mixture was incubated for 30 min at 4°C with head over head mixing. Next, the sample was centrifuged at 4000g and the supernatant was collected as the whole brain cell lysate. Co-immunoprecipitations were performed using protein G dynabeads. First, an in house made RyR2 specific antibody, based on the epitope described in [1], or an IgG negative control antibody were immobilized by incubating 5 µg of the antibody with 20µl of the dynabeads beads in immobilization buffer (PBS + 0.02% tween 20) for at least 30 min at room temperature with head over head mixing. After immobilization the beads were separated from the sample using a magnet followed by one wash with immobilization buffer. Next, the beads were incubated overnight at 4°C with head over head mixing with 800 µg protein of whole brain cell lysate. The next day, the beads were separated from the sample followed by two washes with lysis buffer. Finally, the immunocomplexes were eluted by incubating the beads 50 mM Tris, pH 8, with SDS 0,2% and tween-20 0,1% for 30 minutes at room temperature. NuPAGE LDS Sample Buffer (Thermo Fisher Scientific) was added prior to heating the samples at 70°C for 5 min and subsequent immunoblot analysis. Immunoblot analysis and immunostaining was performed using rabbit polyclonal anti-WFS1 (Thermo Fisher Scientific, PA5-76065), rabbit polyclonal anti-CISD2 (Abclonal, A5231) or mouse monoclonal anti-RyR2 (Thermo Fisher Scientific, MA3-916) for detection[2].

Single-cell expression analysis

Primary cortical neurons were transfected with scrambled, Wfs1, or Cisd2 shRNA encoding plasmids and neuronal transfection marker, Camk2-Ace-8aa-mScarlet at DIV 2-3. The cells were fixed 5 days later with 4% PFA, 5% sucrose prepared in Neurobasal Medium for approximately 12 mins at 37°C, permeabilized with 0.3% Triton X-100 prepared in DPBS for 5 mins at RT, blocked with 5% normal goat serum prepared in DPBS for 1 hour at RT, and incubated overnight with respective primary antibodies at 4°C in a humidity chamber (mouse anti-RYR2 (DSHB, 34C; 1:20), rabbit anti-IP3R1 (Proteintech,19962-1-AP; 1:200), rabbit anti-SERCA2 (Proteintech, 27311-1-AP; 1:200)). Subsequently, the neurons were incubated with secondary antibodies for 1 hour at RT (goat anti-rabbit (Invitrogen, A32731; 1:1000), goat anti-mouse (Invitrogen, A-11001; 1:1000) and visualized under LSM980 confocal microscope under 63X/1.20W lens magnification and 0.8 scan zoom. The

immunofluorescence of RYR2, IP3R1, or SERCA2 in Camk2-Ace-8aa-mScarlet positive cells was further normalized to the immunofluorescence of non-transfected cells to minimize dish-to-dish and experiment-to-experiment variation.

In situ proximity ligation assay

Primary cortical neurons were fixed using 4% paraformaldehyde in a medium containing 5% sucrose for 10 min at 37°C and permeabilized using 1% Triton X-100 in PBS for 7 min at room temperature with constant movement. The cells were then blocked with 10% goat serum and 3% BSA in PBS for 1 h at room temperature and incubated overnight with 1:1000 rabbit anti-FLAG (Sigma-Aldrich) and 1:500 mouse anti-MYC (ThermoFisher Scientific) at 4 °C for overnight. The cells were then washed with 0.1% Tween in PBS, and WFS1 - CISD2 interaction was visualized using Duolink in Situ PLA reagents from Olink Bioscience according to the manufacturer's protocol. All incubations were performed in a humidity chamber at 37°C. First, the samples were incubated with anti-rabbit PLUS 100 and anti-mouse MINUS 100 PLA probes for 1 h followed by ligation and oligonucleotide hybridization for 30 min. Subsequently, an amplification step using red fluorophore-labeled oligonucleotides (excitation maxima 594 nm, emission maxima 624 nm) for 100 min enabled the visualization of the protein interaction as red dots under the confocal microscope. Negative controls were performed using only one of the primary antibodies.

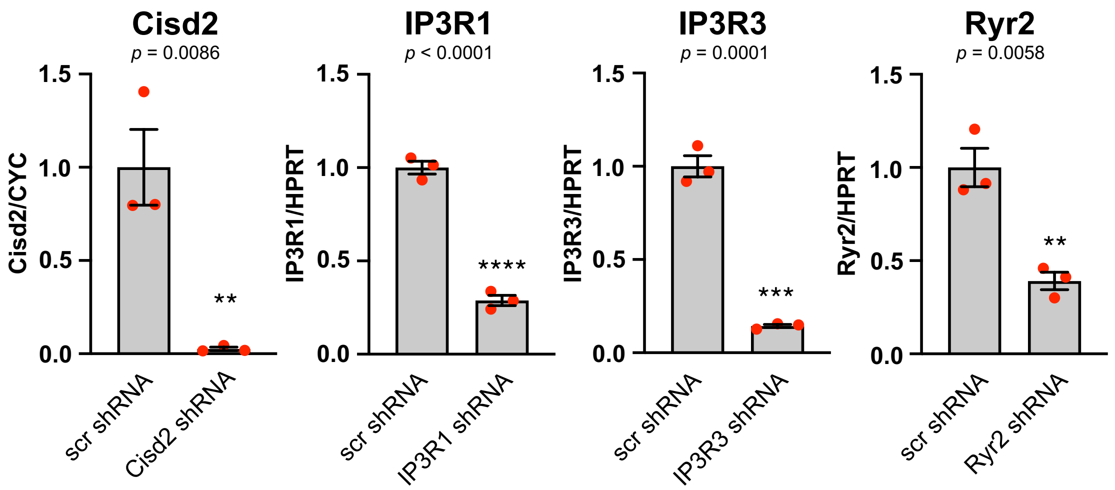

Supplementary Fig. 1. shRNAs against *Cisd2*, *IP$_3$R1, IP$_3$R3*, and *RYR2* efficiently suppress the expression of respective mRNAs.

PC6 cells were transfected with either scrambled shRNA or specific shRNAs and selected for 7 days with 200 µg/ml G418. Total RNA was extracted using RNAeasy mini kit (Qiagen), and first-strand synthesis was performed using 5 µg of total RNA with Maxima First Strand cDNA Synthesis Kit (Thermo). cDNAs were subjected to qPCR using specific primers for CYC or HPRT (housekeeping gene), and using a QuantStudio 12K Flex from Applied Biosystems by Life Technologies. Acquired data were analyzed using the delta Ct method and normalized to transfection efficiency, which was estimated separately. n = 3 independent dishes, two-tailed unpaired t-test.

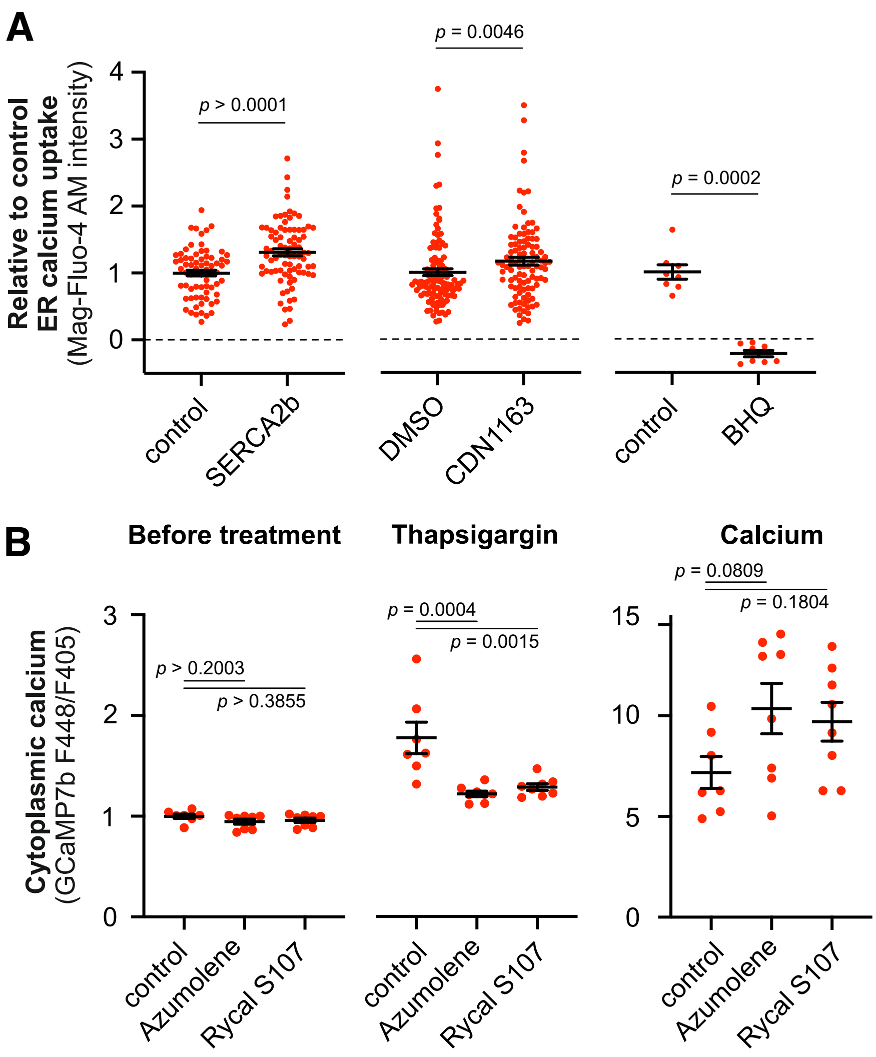

Supplementary Fig. 2. Effect of SERCA activators and inhibitor on ER Ca^{2+} uptake and Ryanodine receptor inhibitors on ER Ca^{2+} release.

(A) ER Ca^{2+} uptake is affected in outer-membrane permeabilized SERCA2b overexpressing or CDN1163 or BHQ treated neurons. Outer membrane permeabilized neurons were stained with Mag-Fluo-4 AM and treated with sodium azide to inhibit mitochondrial Ca^{2+} uptake. Following treatment with CDN1163 (2 µM) or 2,5-di-t-butyl-1,4-benzohydroquinone (BHQ, 2 µM) the free Ca^{2+} concentration was then increased from pCa9 to pCa6.5 by adding $CaCl_2$. Data are presented as relative Ca^{2+} uptake of transfected or treated cell to Ca^{2+} uptake in non-transfected or untreated neurons. *n* = 69 and 81 (control and SERCA2b), 115 and 101 (control and CDN1162) and 8 and 6 (control and BHQ) neurons, two-tailed Mann-Whitney test.

(B) Thapsigargin-induced Ca^{2+} release from the ER to the cytoplasm is lower in azumolene (20 µM) or Rycal S107 (5 µM) treated neurons. (48 h pretreatment). The left panel depicts the Ca^{2+} transient obtained after treatment with 2 µM thapsigargin in Ca^{2+}-free media in the presence of 0.5 mM EGTA and the effect of subsequent reintroduction of external 10 mM Ca^{2+}. The left and middle panels show the cytosolic Ca^{2+} levels before and after the thapsigargin-induced transients (2 µM thapsigargin in Ca^{2+}-free media), and the right panel shows the effect of reintroduced Ca^{2+}. The Ca^{2+} measurements were performed in a ratiometric mode allowing direct comparison of the values between the groups. *n* = 7 (control) or 8 (azumolene and Rycal S107 groups) neurons. Brown-Forsythe ANOVA and Dunnett's T3 multiple comparisons test.

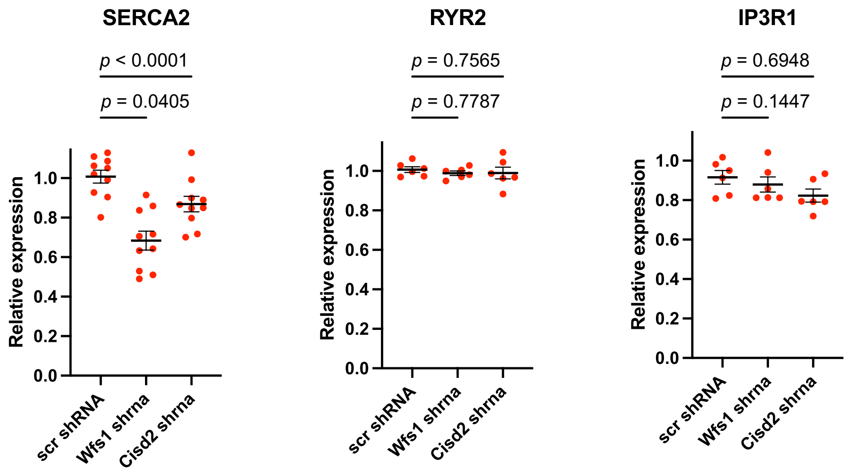

Supplementary Fig. 3. Single-cell expression analysis of SERCA2, RYR2, and IP3R1 in Wfs1 or Cisd2 knockdown neurons. Primary cortical neurons were transfected with scrambled, Wfs1, or Cisd2 shRNA encoding plasmids and neuronal transfection marker, Camk2-Ace-8aa-mScarlet at DIV 2-3, fixed 5 days later and stained with anti-SERCA2, anti-RYR2 or anti-IP3R1 antibodies. The immunofluorescence of RYR2, IP3R1, or SERCA2 in Camk2-Ace-8aa-mScarlet positive cells was further normalized to the immunofluorescence of non-transfected cells to minimize dish-to-dish and experiment-to-experiment variation. n = 9 (SERCA) or 6 (RYR2 and IP3R1) neurons, One-way ANOVA and Dunnett's multiple comparisons test.

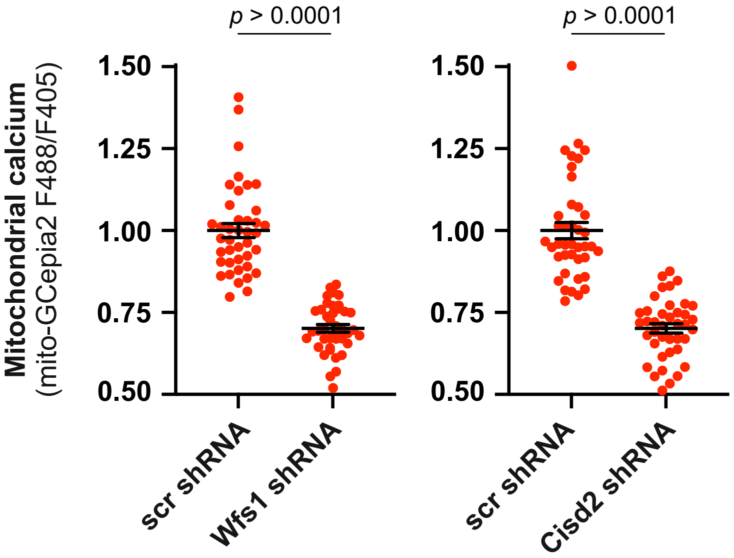

Supplementary Fig. 4. Decreased resting mitochondrial Ca^{2+} levels in the axons of WFS1 and CISD2 deficient neurons as measured by higher affinity mitochondria-targeted Ca^{2+} indicator GCepia2. n = 40 neurons, two-tailed Mann-Whitney test.

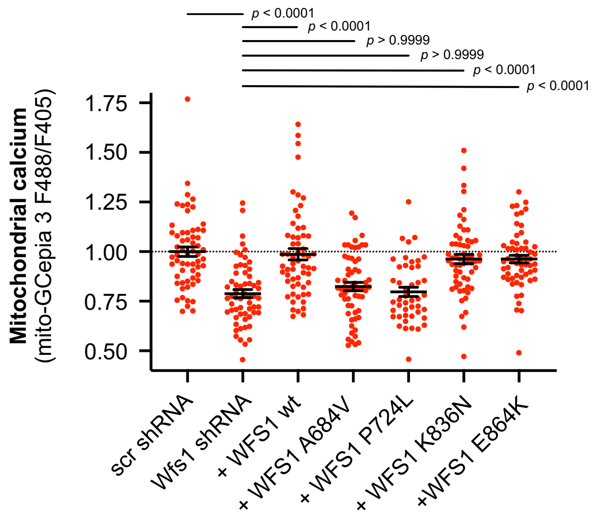

Supplementary Fig. 5. Wt, K836N and 864K, but not A684V and P724L WFS1, were able to restore resting mitochondrial Ca^{2+} levels in the axons of WFS1-deficient neurons. *n* = 60, 60, 59, 60, 45, 59 and 60 neurons, Kruskal-Wallis test and Dunn's multiple comparisons test.

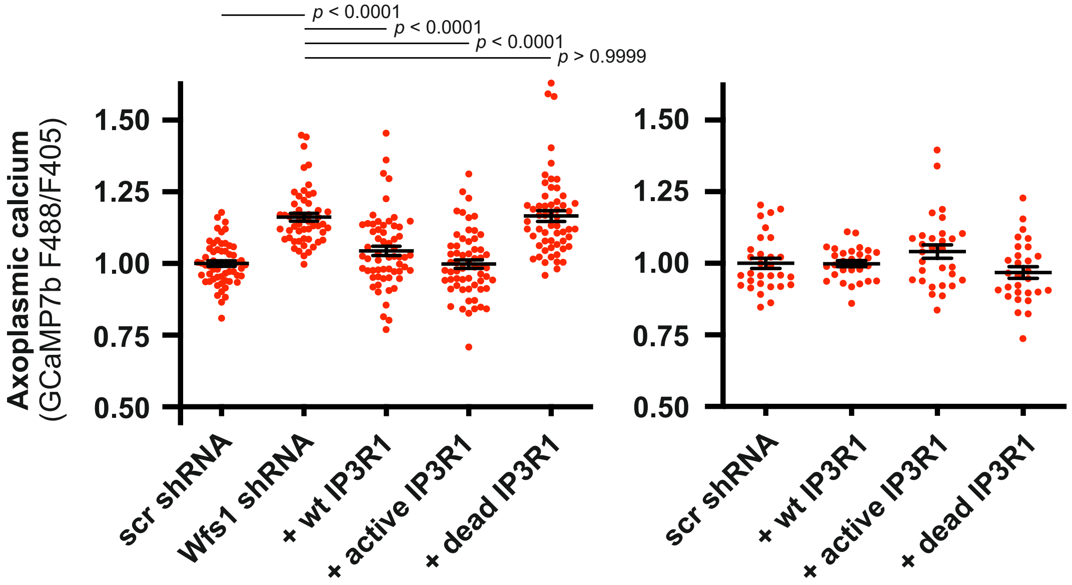

Supplementary Fig. 6. Overexpression of IP_3R1 but not its pore-dead mutant restores cytosolic Ca^{2+} levels in the axons of WFS1 deficient neurons. *n* = 59, 53, 60, 60 and 57 neurons (left panel), Kruskal-Wallis test and Dunn's multiple comparisons test. *n* = 30, 29, 30 and 29 neurons (right panel), Brown-Forsythe ANOVA and Dunnett's T3 multiple comparisons test.

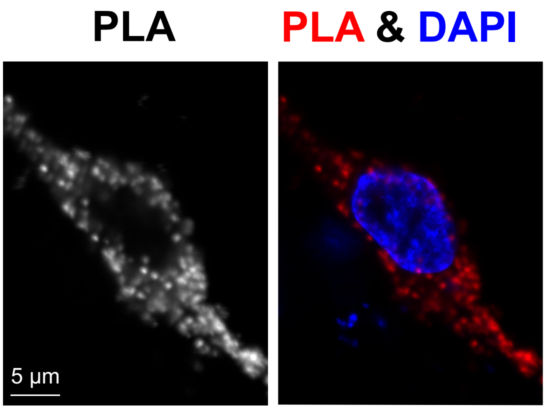

Supplementary Figure 7. Proximity Ligation Assay (PLA) reveals close proximity of WFS1 and CISD2 in neurons.

Primary cortical neurons transfected with WFS1-myc and CISD2-flag were fixed using 4% paraformaldehyde in a medium containing 5% sucrose for 10 min at 37°C and permeabilized using 1% Triton X-100 in PBS for 7 min at room temperature with constant movement. The cells were then blocked with 10% goat serum and 3% BSA in PBS for 1 h at room temperature and incubated overnight with 1:1000 rabbit anti-FLAG (Sigma-Aldrich) and 1:500 mouse anti-MYC (ThermoFisher Scientific) at 4 °C for overnight. The cells were then washed with 0.1% Tween in PBS, and WFS1 - CISD2 interaction was visualized using Duolink in Situ PLA reagents from Olink Bioscience according to the manufacturer's protocol. All incubations were performed in a humidity chamber at 37°C. First, the samples were incubated with anti-rabbit PLUS 100 and anti-mouse MINUS 100 PLA probes for 1 h followed by ligation and oligonucleotide hybridization for 30 min. Subsequently, an amplification step using red fluorophore-labeled oligonucleotides (excitation maxima 594 nm, emission maxima 624nm) for 100 min enabled the visualization of the protein interaction as red dots under the confocal microscope. Negative controls were performed using only one of the primary antibodies.

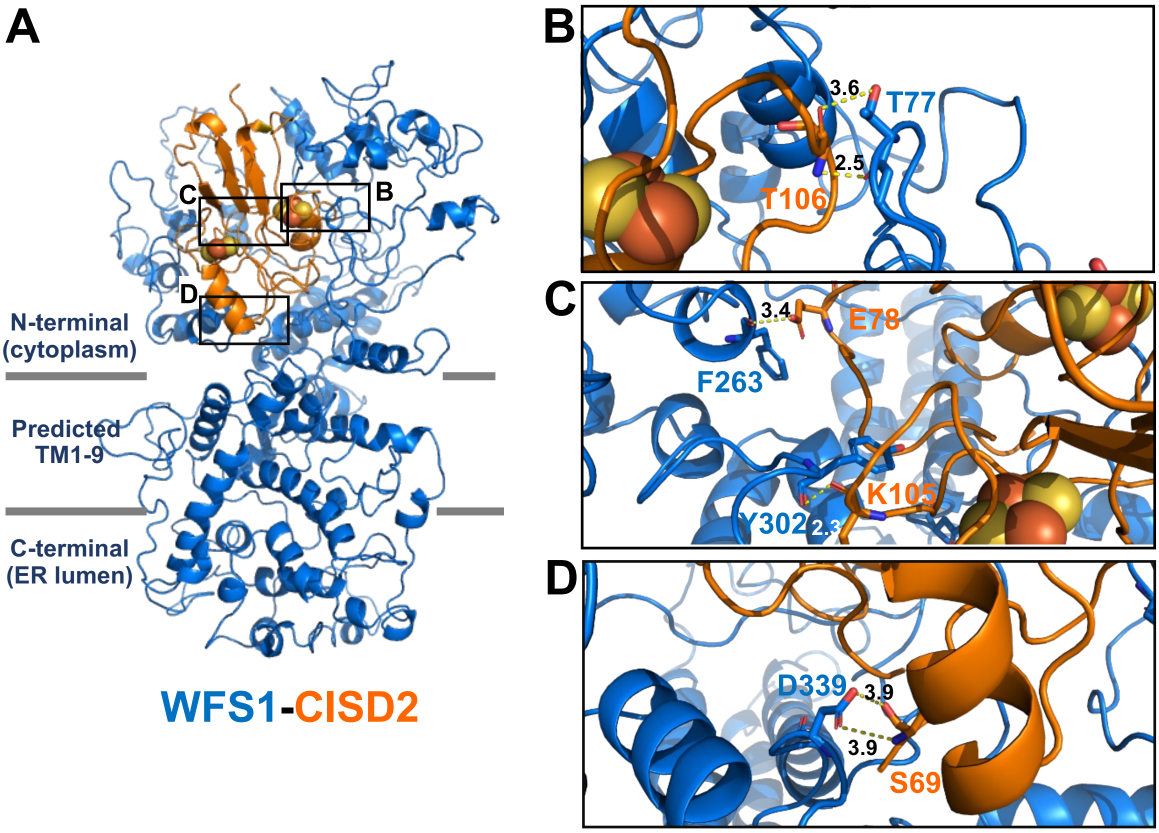

Supplementary Fig. 8. Computational docking model for human CISD2 and WFS1. (A) ZDOCK web server (http://zdock.umassmed.edu/) was used to perform protein-protein molecular docking. The ZDOCK score for the CISD2/WFS1 docking model was 1430.867. The crystal structure of CISD2 (orange) was available in Protein Data Bank (PDB ID: 3FNV). The predicted structure and transmembrane domains of WFS1 (blue) were obtained from Qian et al. 2015[3]. Magnified views of WFS1-CISD2 interactions were shown in (B)-(D) to demonstrate the potential hydrogen bonds formed between WFS1 and CISD2 (yellow lines).

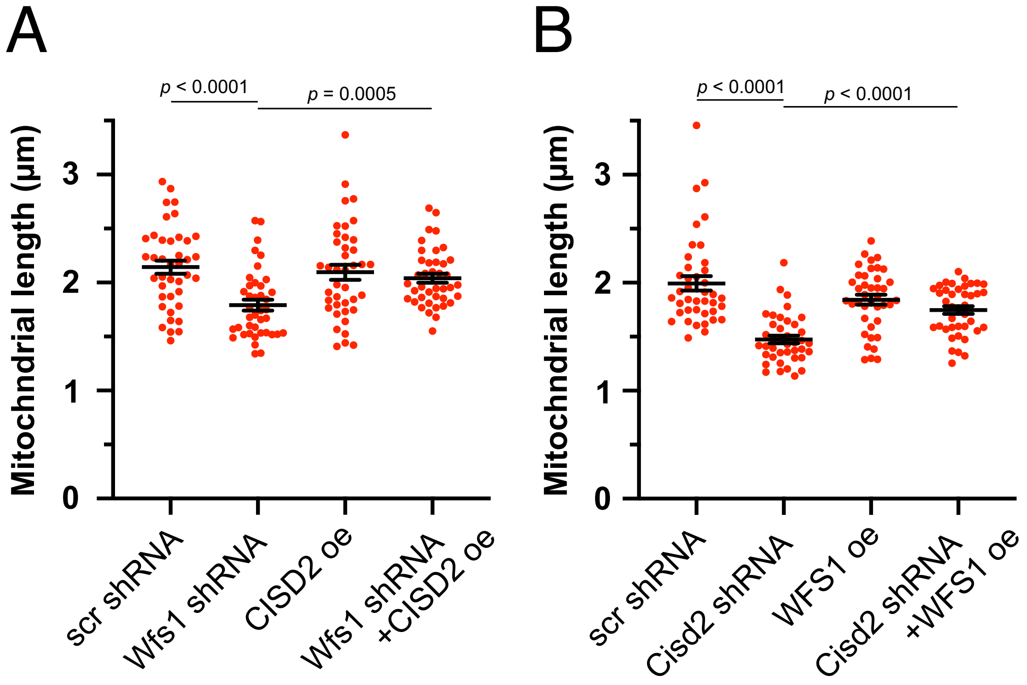

Supplementary Fig. 9. Mitochondrial length is restored in WFS1-deficient neurons when overexpressing CISD2 (A) and in CISD2-deficient neurons when overexpressing WFS1 (B). n = 40 neurons, Brown-Forsythe ANOVA and Dunn's multiple comparisons test (A). n = 40 neurons, Kruskal-Wallis test and Dunn's multiple comparisons test (B).

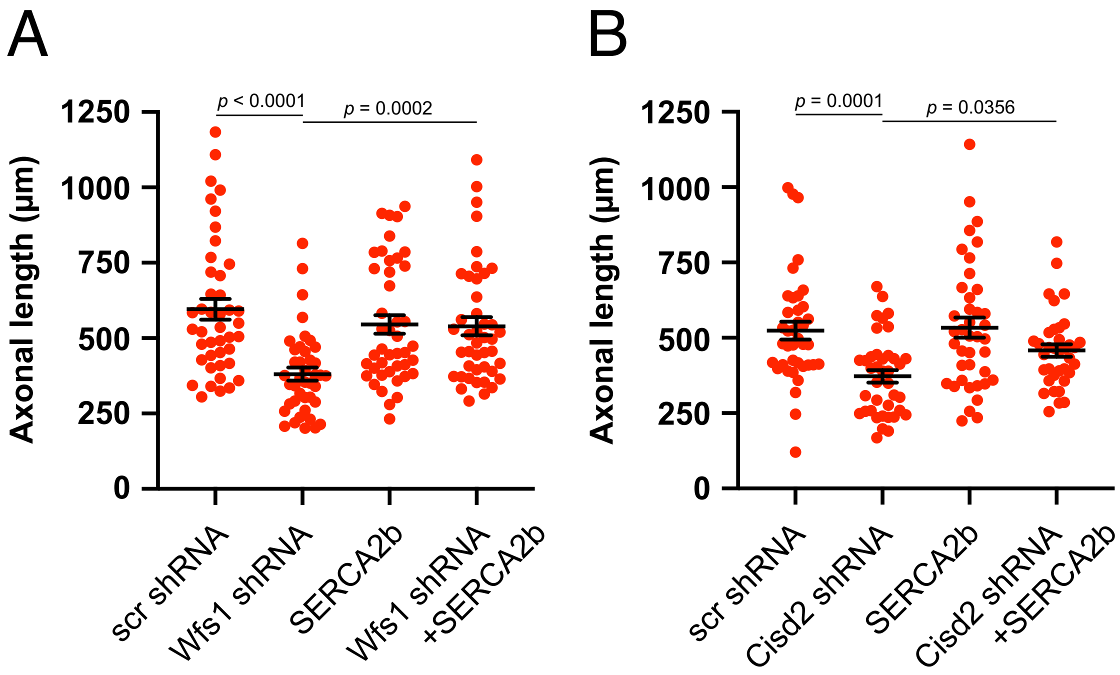

Supplementary Fig. 10. Suppressed axonal growth in WFS1- (A) or CISD2-deficient (B) neurons is restored when expressing SERCA2b. n = 43, 41, 43 ad 43 neurons (A), n = 39, 39, 40, 36 neurons (B), both Kruskal-Wallis test and Dunn's multiple comparisons test.

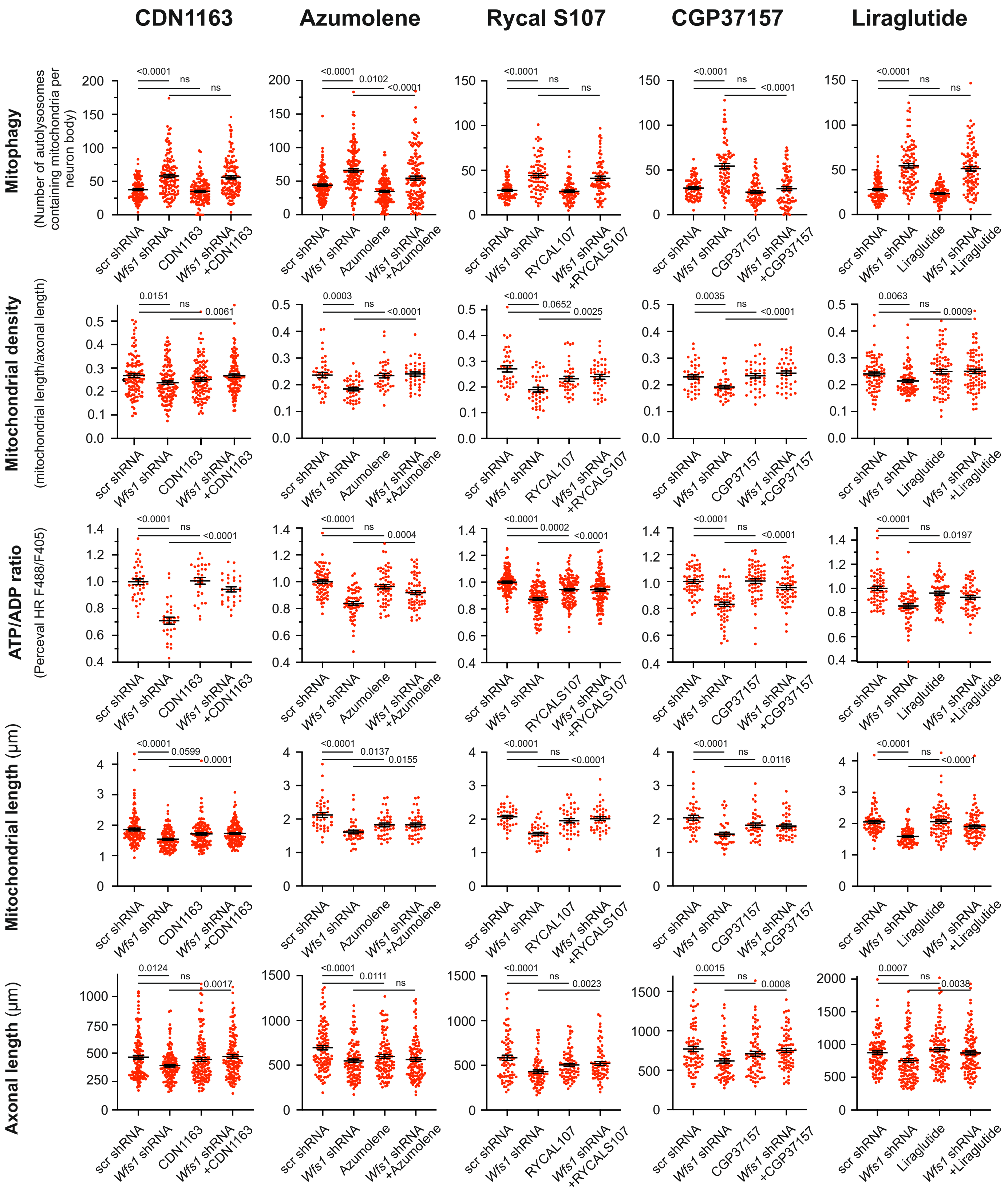

Supplementary Fig. 11. Effects of different compounds on mitophagy, mitochondrial density, ATP/ADP ratio, mitochondrial length, and axonal development. *n* values are given in Source Data for Supplementary Figures. One-way ANOVA and Šídák's multiple comparisons test, Brown-Forsythe ANOVA and Dunn's multiple comparisons test, Kruskal-Wallis test and Dunn's multiple comparisons test.

Supplementary References

1. Lacampagne, A. *et al.* Post-translational remodeling of ryanodine receptor induces calcium leak leading to Alzheimer's disease-like pathologies and cognitive deficits. *Acta Neuropathol* **134**, 749-767 (2017). https://doi.org/10.1007/s00401-017-1733-7
2. Vervliet, T. *et al.* Bcl-2 binds to and inhibits ryanodine receptors. *J Cell Sci* **127**, 2782-2792 (2014). https://doi.org/10.1242/jcs.150011
3. Qian, X., Qin, L., Xing, G. & Cao, X. Phenotype Prediction of Pathogenic Nonsynonymous Single Nucleotide Polymorphisms in WFS1. *Sci Rep* **5**, 14731 (2015). https://doi.org/10.1038/srep14731
