# Supplementary material for: ER calcium depletion as a key driver for impaired ER-to-mitochondria calcium transfer and mitochondrial dysfunction in Wolfram syndrome
Source: Nat Commun. 2024 Jul 21;15:6143. doi: 10.1038/s41467-024-50502-x (PMC11271478; doi:10.1038/s41467-024-50502-x)
Supplement: Supplementary file 3 — Reporting Summary [file 41467_2024_50502_MOESM3_ESM.pdf]

nature portfolio

Corresponding author(s): prof. Allen Kaasik
Dr. Annika Vaarmann

Last updated by author(s): Apr 29, 2024

Reporting Summary

Nature Portfolio wishes to improve the reproducibility of the work that we publish. This form provides structure for consistency and transparency in reporting. For further information on Nature Portfolio policies, see our Editorial Policies and the Editorial Policy Checklist.

Statistics

For all statistical analyses, confirm that the following items are present in the figure legend, table legend, main text, or Methods section.

n/a	Confirmed	
☐	☒	The exact sample size (*n*) for each experimental group/condition, given as a discrete number and unit of measurement
☐	☒	A statement on whether measurements were taken from distinct samples or whether the same sample was measured repeatedly
☐	☒	The statistical test(s) used AND whether they are one- or two-sided<br>*Only common tests should be described solely by name; describe more complex techniques in the Methods section.*
☒	☐	A description of all covariates tested
☐	☒	A description of any assumptions or corrections, such as tests of normality and adjustment for multiple comparisons
☐	☒	A full description of the statistical parameters including central tendency (e.g. means) or other basic estimates (e.g. regression coefficient) AND variation (e.g. standard deviation) or associated estimates of uncertainty (e.g. confidence intervals)
☒	☐	For null hypothesis testing, the test statistic (e.g. *F*, *t*, *r*) with confidence intervals, effect sizes, degrees of freedom and *P* value noted<br>*Give P values as exact values whenever suitable.*
☒	☐	For Bayesian analysis, information on the choice of priors and Markov chain Monte Carlo settings
☒	☐	For hierarchical and complex designs, identification of the appropriate level for tests and full reporting of outcomes
☒	☐	Estimates of effect sizes (e.g. Cohen's *d*, Pearson's *r*), indicating how they were calculated

Our web collection on statistics for biologists contains articles on many of the points above.

Software and code

Policy information about availability of computer code

Data collection	Zeiss ZEN black 2.1 SP1<br>Zeiss ZEN blue 3.1 software<br>Zeiss LSM 510 software (Carl Zeiss Microscopy GmbH, Germany)<br>Olympus CellSens entry software (version 2.2, Olympus, Japan)<br>Image Studio Ver 2.0 Ink (LICOR Biosciences)<br>QuantStudio 12K Flex Real-Time PCR System (Applied Biosystems by Life technologies)<br>Instinct Software (Promega, USA)
Data analysis	MicroImage software (Media Cybernetics, Bethesda, MD)<br>Fiji and Fiji plugin NeuronJ

For manuscripts utilizing custom algorithms or software that are central to the research but not yet described in published literature, software must be made available to editors and reviewers. We strongly encourage code deposition in a community repository (e.g. GitHub). See the Nature Portfolio guidelines for submitting code & software for further information.

Data

Policy information about availability of data

All manuscripts must include a data availability statement. This statement should provide the following information, where applicable:

- Accession codes, unique identifiers, or web links for publicly available datasets
- A description of any restrictions on data availability
- For clinical datasets or third party data, please ensure that the statement adheres to our policy

We have employed scatter graphs to depict the predominant portion of the data. At present, the original numerical data is not incorporated; however, in the subsequent phase, we intend to include it as supplementary data and will provide respective data availability statement.

Research involving human participants, their data, or biological material

Policy information about studies with human participants or human data. See also policy information about sex, gender (identity/presentation), and sexual orientation and race, ethnicity and racism.

Reporting on sex and gender	na
Reporting on race, ethnicity, or other socially relevant groupings	na
Population characteristics	na
Recruitment	na
Ethics oversight	na

Note that full information on the approval of the study protocol must also be provided in the manuscript.

Field-specific reporting

Please select the one below that is the best fit for your research. If you are not sure, read the appropriate sections before making your selection.

☒ Life sciences ☐ Behavioural & social sciences ☐ Ecological, evolutionary & environmental sciences

For a reference copy of the document with all sections, see nature.com/documents/nr-reporting-summary-flat.pdf

Life sciences study design

All studies must disclose on these points even when the disclosure is negative.

Sample size	The requisite sample size was empirically determined, relying on our prior experience with the employed imaging methods. This estimation is considered adequate for detecting subtle differences in the data.
Data exclusions	In the case of calcium experiments, outliers were removed (ROUT, Q 1%) to exclude bursting neurons.
Replication	All key experiments were repeated at least twice and were reproducible.
Randomization	In microscopy experiments, individual dishes were randomly allocated to the experimental groups.
Blinding	Blinding was implemented in microscopy image analysis when the human factor in analysis was deemed relevant. In cases where it did not impact image analysis, blinding procedures were not applied.

Reporting for specific materials, systems and methods

We require information from authors about some types of materials, experimental systems and methods used in many studies. Here, indicate whether each material, system or method listed is relevant to your study. If you are not sure if a list item applies to your research, read the appropriate section before selecting a response.

Materials & experimental systems

n/a	Involved in the study
☐	☒ Antibodies
☐	☒ Eukaryotic cell lines
☐	☐ Palaeontology and archaeology
☐	☒ Animals and other organisms
☐	☐ Clinical data
☐	☐ Dual use research of concern
☐	☐ Plants

Methods

n/a	Involved in the study
☐	☐ ChIP-seq
☐	☐ Flow cytometry
☐	☐ MRI-based neuroimaging

Antibodies

Antibodies used

Rabbit polyclonal anti-WFS1 (1:1000) Proteintech 26995-1-AP
Rabbit polyclonal anti-WFS1 (1:1000) Thermo Fisher Scientific PA5-76065
Mouse monoclonal anti-CISD2, clone 3D7A3 (1:1000) Proteintech 66082-1-Ig
Rabbit polyclonal anti-CISD2 (1:1000) Proteintech 13318-1-AP
Rabbit polyclonal anti-CISD2 (1:1000) Abclonal A5231
Rabbit polyclonal anti-SERCA2 (1:1000 WB, 1:200 IC) Proteintech 27311-1-AP
Mouse monoclonal anti-SERCA2, clone IID8 (1:1000) Invitrogen MA3-910
Mouse monoclonal anti-RYR, clone 34C (1:20 IC) DSHB 34C
Mouse monoclonal anti-RyR2, clone C3-33 (1:1000) Thermo Fisher Scientific MA3-916
Rabbit polyclonal anti-RyR2 (1:1000) Generated in our lab
Rabbit polyclonal anti-IP3R (1:200 IC) Proteintech 19962-1-AP
Mouse monoclonal anti-β-actin, clone AC-74 (1:2000) Sigma-Aldrich A228
Rabbit anti-MYC (1:3000) Abcam ab9106
Rabbit anti-FLAG (1:1000) Sigma-Aldrich F7425
Mouse anti-MYC (1:500) Thermo Fisher Scientific R950-25
Mouse anti-GFP (1:1000) Roche 11814460001
Goat anti-Rabbit Alexa Fluor™ Plus 488 (1:1000 IC) Invitrogen A32731
Goat anti-Mouse Alexa Fluor™ 488 (1:1000 IC) Invitrogen A11001
Goat anti-mouse IRDye 680LT (1:2000) LI-COR Biosciences 926-68020
Goat anti-mouse IRDye 800CW (1:5000) LI-COR Biosciences 925-32210
Goat anti-rabbit IRDye 800CW (1:5000) LI-COR Biosciences 926-32211
Goat anti-rabbit IRDye 680LT (1:2000) LI-COR Biosciences 926-68021
Donkey anti-mouse MINUS 100 (according to manufacturer) Olink Bioscience 92004-0100
Donkey anti-rabbit PLUS 100 (according to manufacturer) Olink Bioscience 92002-0100

Validation

Based on manufacturers websites:

Rabbit polyclonal anti-WFS1 (Proteintech 26995-1-AP) is validated for Human, Mouse, Rat (KD/KO validated) for WB, IF, IHC, ELISA
Rabbit polyconal anti-WFS1 ThermoFisher Scientific PA5-76065 is validated for Human and Mouse for WB, IHC and IF.
Mouse monoclonal anti-CISD2 (Proteintech 66082-1-Ig) is validated for Human, Rat, Mouse (KD/KO validated) for WB, IF, IHC, ELISA
Rabbit polyclonal anti-CISD2 (Proteintech 13318-1-AP) is validated for Human, Mouse, Rat (KD/KO validated) for WB, IP, IF, FC, IHC...
Rabbit polyconal anti-CISD2 (Abclonal A5231) is validated for Human, Mouse and Rat for WB, IHC and ELISA.
Rabbit polyclonal anti -SERCA2 (Proteintech 27311-1-AP) is validated for Human, Mouse for WB, IF, IHC, ELISA
Mouse monoclonal anti-SERCA2 (Invitrogen MA3-910) is validated for Bovine, Dog, Human, Mouse, Pig, Rabbit, Rat for WB, IF, IHC
Mouse monoclonal anti-RYR (DSHB 34C) is validated for Human, Mouse, Rabbit for WB, IHC, IF
Mouse monoclonal anti-RyR2 (Thermo Fisher Scientific MA3-916) validated for Human, Mouse and Rat for WB, IHC and IF.
Rabbit polyclonal anti-RyR2 (Generated in our lab) validated Lacampagne et al 2017
Rabbit polyclonal anti-IP3R (Proteintech 19962-1-AP) is validated for Human, Mouse, Rat for WB, IP, FC, IHC, ELISA
Mouse monoclonal anti-β-actin (clone AC-74) Sigma-Aldrich A228-200UL is validated for Human, Mouse and Rat for WB, IHC and IF.
Rabbit anti-MYC Abcam ab9106 is tested for ICC/IF, IP, WB and IHC-P.
Rabbit anti-FLAG Sigma-Aldrich F7425 is tested for IF, IP and WP.
Mouse anti-MYC Thermo Fisher Scientific R950-25 is tested for WB.
Mouse anti-GFP Roche 11814460001 is tested for WB, IP and IF.

Eukaryotic cell lines

Policy information about cell lines and Sex and Gender in Research

Cell line source(s)

HEK293 (ATCC CRL-1573) American Type Culture Collection
PC6-3 (Cellosaurus RRID: CVCL_7101) cell line was gift from Dan Lindholm lab.

Authentication

The HEK293 and PC6 cell lines utilized in this study were not subjected to specific authentication procedures. Nonetheless, their consistent morphology and behavior under the specified experimental conditions have been observed throughout the duration of the study.

Mycoplasma contamination	The MycoStrip™ Mycoplasma Detection Kit has been occasionally employed to eliminate the possibility of mycoplasma contamination.
Commonly misidentified lines (See ICLAC register)	na

Palaeontology and Archaeology

Specimen provenance	*Provide provenance information for specimens and describe permits that were obtained for the work (including the name of the issuing authority, the date of issue, and any identifying information). Permits should encompass collection and, where applicable, export.*
Specimen deposition	*Indicate where the specimens have been deposited to permit free access by other researchers.*
Dating methods	*If new dates are provided, describe how they were obtained (e.g. collection, storage, sample pretreatment and measurement), where they were obtained (i.e. lab name), the calibration program and the protocol for quality assurance OR state that no new dates are provided.*

☐ Tick this box to confirm that the raw and calibrated dates are available in the paper or in Supplementary Information.

Ethics oversight	*Identify the organization(s) that approved or provided guidance on the study protocol, OR state that no ethical approval or guidance was required and explain why not.*

Note that full information on the approval of the study protocol must also be provided in the manuscript.

Animals and other research organisms

Policy information about studies involving animals; ARRIVE guidelines recommended for reporting animal research, and Sex and Gender in Research

Laboratory animals	Primary cultures of rat cortical neurons were prepared from < 1-day-old neonatal Wistar rats.
Wild animals	na
Reporting on sex	na
Field-collected samples	na
Ethics oversight	The research in this manuscript doesn't involve procedures that require ethical authorization under the Estonian 'Animal Protection Act,' Chapter 8.<br>The project involves only the preparation of primary neuronal cultures, and no experiments are performed on live animals.<br>We also certify that the animals used in the project will be bred, kept, and euthanized at the Laboratory Animal Centre according to the Estonian Animal Protection Act and under the activity license given by the Estonian Veterinary and Food Board.

Note that full information on the approval of the study protocol must also be provided in the manuscript.

Clinical data

Policy information about clinical studies

All manuscripts should comply with the ICMJE guidelines for publication of clinical research and a completed CONSORT checklist must be included with all submissions.

Clinical trial registration	*Provide the trial registration number from ClinicalTrials.gov or an equivalent agency.*
Study protocol	*Note where the full trial protocol can be accessed OR if not available, explain why.*
Data collection	*Describe the settings and locales of data collection, noting the time periods of recruitment and data collection.*
Outcomes	*Describe how you pre-defined primary and secondary outcome measures and how you assessed these measures.*

Dual use research of concern

Policy information about dual use research of concern

Hazards

Could the accidental, deliberate or reckless misuse of agents or technologies generated in the work, or the application of information presented in the manuscript, pose a threat to:

No	Yes	
☒	☐	Public health
☒	☐	National security
☒	☐	Crops and/or livestock
☒	☐	Ecosystems
☒	☐	Any other significant area

Experiments of concern

Does the work involve any of these experiments of concern:

No	Yes	
☒	☐	Demonstrate how to render a vaccine ineffective
☒	☐	Confer resistance to therapeutically useful antibiotics or antiviral agents
☒	☐	Enhance the virulence of a pathogen or render a nonpathogen virulent
☒	☐	Increase transmissibility of a pathogen
☒	☐	Alter the host range of a pathogen
☒	☐	Enable evasion of diagnostic/detection modalities
☒	☐	Enable the weaponization of a biological agent or toxin
☒	☐	Any other potentially harmful combination of experiments and agents

Plants

Seed stocks	*Report on the source of all seed stocks or other plant material used. If applicable, state the seed stock centre and catalogue number. If plant specimens were collected from the field, describe the collection location, date and sampling procedures.*
Novel plant genotypes	*Describe the methods by which all novel plant genotypes were produced. This includes those generated by transgenic approaches, gene editing, chemical/radiation-based mutagenesis and hybridization. For transgenic lines, describe the transformation method, the number of independent lines analyzed and the generation upon which experiments were performed. For gene-edited lines, describe the editor used, the endogenous sequence targeted for editing, the targeting guide RNA sequence (if applicable) and how the editor was applied.*
Authentication	*Describe any authentication procedures for each seed stock used or novel genotype generated. Describe any experiments used to assess the effect of a mutation and, where applicable, how potential secondary effects (e.g. second site T-DNA insertions, mosiacism, off-target gene editing) were examined.*

ChIP-seq

Data deposition

☐ Confirm that both raw and final processed data have been deposited in a public database such as GEO.

☐ Confirm that you have deposited or provided access to graph files (e.g. BED files) for the called peaks.

Data access links<br>*May remain private before publication.*	*For "Initial submission" or "Revised version" documents, provide reviewer access links. For your "Final submission" document, provide a link to the deposited data.*
Files in database submission	*Provide a list of all files available in the database submission.*
Genome browser session<br>(e.g. UCSC)	*Provide a link to an anonymized genome browser session for "Initial submission" and "Revised version" documents only, to enable peer review. Write "no longer applicable" for "Final submission" documents.*

Methodology

Replicates	*Describe the experimental replicates, specifying number, type and replicate agreement.*
Sequencing depth	*Describe the sequencing depth for each experiment, providing the total number of reads, uniquely mapped reads, length of reads and whether they were paired- or single-end.*
Antibodies	*Describe the antibodies used for the ChIP-seq experiments; as applicable, provide supplier name, catalog number, clone name, and lot number.*

Peak calling parameters	*Specify the command line program and parameters used for read mapping and peak calling, including the ChIP, control and index files used.*
Data quality	*Describe the methods used to ensure data quality in full detail, including how many peaks are at FDR 5% and above 5-fold enrichment.*
Software	*Describe the software used to collect and analyze the ChIP-seq data. For custom code that has been deposited into a community repository, provide accession details.*

Flow Cytometry

Plots

Confirm that:

☐ The axis labels state the marker and fluorochrome used (e.g. CD4-FITC).

☐ The axis scales are clearly visible. Include numbers along axes only for bottom left plot of group (a 'group' is an analysis of identical markers).

☐ All plots are contour plots with outliers or pseudocolor plots.

☐ A numerical value for number of cells or percentage (with statistics) is provided.

Methodology

Sample preparation	*Describe the sample preparation, detailing the biological source of the cells and any tissue processing steps used.*
Instrument	*Identify the instrument used for data collection, specifying make and model number.*
Software	*Describe the software used to collect and analyze the flow cytometry data. For custom code that has been deposited into a community repository, provide accession details.*
Cell population abundance	*Describe the abundance of the relevant cell populations within post-sort fractions, providing details on the purity of the samples and how it was determined.*
Gating strategy	*Describe the gating strategy used for all relevant experiments, specifying the preliminary FSC/SSC gates of the starting cell population, indicating where boundaries between "positive" and "negative" staining cell populations are defined.*

☐ Tick this box to confirm that a figure exemplifying the gating strategy is provided in the Supplementary Information.

Magnetic resonance imaging

Experimental design

Design type	*Indicate task or resting state; event-related or block design.*
Design specifications	*Specify the number of blocks, trials or experimental units per session and/or subject, and specify the length of each trial or block (if trials are blocked) and interval between trials.*
Behavioral performance measures	*State number and/or type of variables recorded (e.g. correct button press, response time) and what statistics were used to establish that the subjects were performing the task as expected (e.g. mean, range, and/or standard deviation across subjects).*

Acquisition

Imaging type(s)	*Specify: functional, structural, diffusion, perfusion.*
Field strength	*Specify in Tesla*
Sequence & imaging parameters	*Specify the pulse sequence type (gradient echo, spin echo, etc.), imaging type (EPI, spiral, etc.), field of view, matrix size, slice thickness, orientation and TE/TR/flip angle.*
Area of acquisition	*State whether a whole brain scan was used OR define the area of acquisition, describing how the region was determined.*

Diffusion MRI ☐ Used ☐ Not used

Preprocessing

Preprocessing software	*Provide detail on software version and revision number and on specific parameters (model/functions, brain extraction, segmentation, smoothing kernel size, etc.).*

Normalization | *If data were normalized/standardized, describe the approach(es): specify linear or non-linear and define image types used for transformation OR indicate that data were not normalized and explain rationale for lack of normalization.*

Normalization template | *Describe the template used for normalization/transformation, specifying subject space or group standardized space (e.g. original Talairach, MNI305, ICBM152) OR indicate that the data were not normalized.*

Noise and artifact removal | *Describe your procedure(s) for artifact and structured noise removal, specifying motion parameters, tissue signals and physiological signals (heart rate, respiration).*

Volume censoring | *Define your software and/or method and criteria for volume censoring, and state the extent of such censoring.*

Statistical modeling & inference

Model type and settings | *Specify type (mass univariate, multivariate, RSA, predictive, etc.) and describe essential details of the model at the first and second levels (e.g. fixed, random or mixed effects; drift or auto-correlation).*

Effect(s) tested | *Define precise effect in terms of the task or stimulus conditions instead of psychological concepts and indicate whether ANOVA or factorial designs were used.*

Specify type of analysis: ☐ Whole brain ☐ ROI-based ☐ Both

Statistic type for inference | *Specify voxel-wise or cluster-wise and report all relevant parameters for cluster-wise methods.*

(See Eklund et al. 2016)

Correction | *Describe the type of correction and how it is obtained for multiple comparisons (e.g. FWE, FDR, permutation or Monte Carlo).*

Models & analysis

n/a	Involved in the study
☐	☐ Functional and/or effective connectivity
☐	☐ Graph analysis
☐	☐ Multivariate modeling or predictive analysis

Functional and/or effective connectivity | *Report the measures of dependence used and the model details (e.g. Pearson correlation, partial correlation, mutual information).*

Graph analysis | *Report the dependent variable and connectivity measure, specifying weighted graph or binarized graph, subject- or group-level, and the global and/or node summaries used (e.g. clustering coefficient, efficiency, etc.).*

Multivariate modeling and predictive analysis | *Specify independent variables, features extraction and dimension reduction, model, training and evaluation metrics.*
