# Supplementary figures and images for: ER calcium depletion as a key driver for impaired ER-to-mitochondria calcium transfer and mitochondrial dysfunction in Wolfram syndrome
Source: Nat Commun. 2024 Jul 21;15:6143. doi: 10.1038/s41467-024-50502-x (PMC11271478; doi:10.1038/s41467-024-50502-x)

Figure 7C

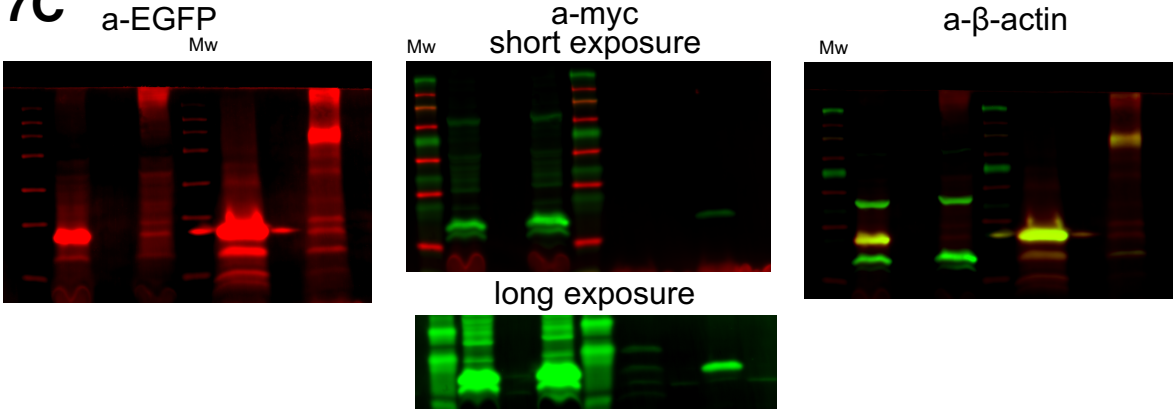

Figure 7D

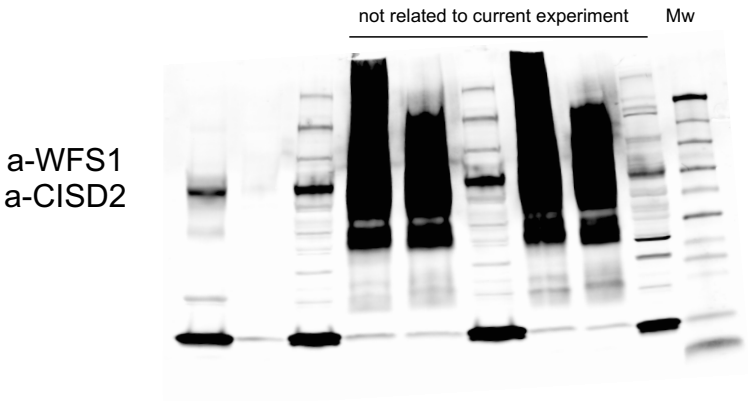

Figure 7E

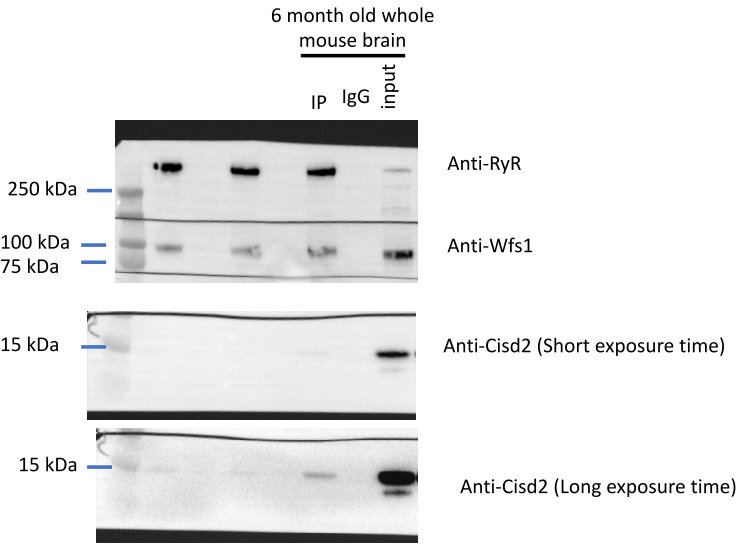

Figure 7F

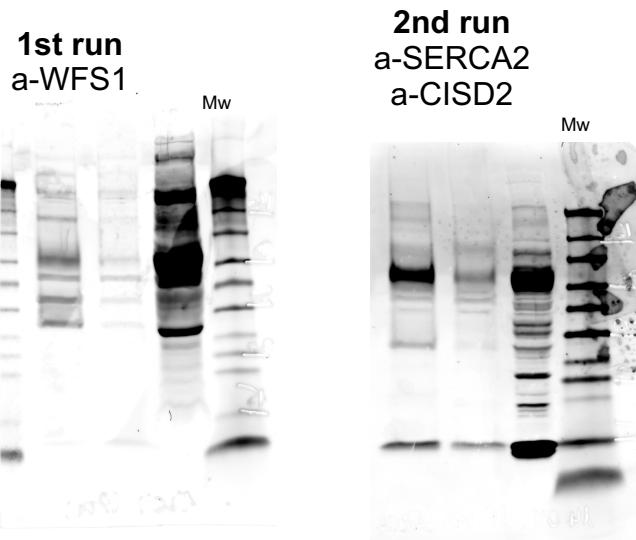

Supplement: Supplementary file 4 — Source Data [file 41467_2024_50502_MOESM4_ESM.zip › Source data Western Blots.pdf]
